# Chemo-Radiotherapy of Oligometastases of Colorectal Cancer With Pegylated Liposomal Mitomycin-C Prodrug (Promitil): Mechanistic Basis and Preliminary Clinical Experience

**DOI:** 10.3389/fonc.2018.00544

**Published:** 2018-11-26

**Authors:** Esther Tahover, Rachel Bar-Shalom, Eli Sapir, Raphael Pfeffer, Igor Nemirovsky, Yehonatan Turner, Maya Gips, Patricia Ohana, Benjamin W. Corn, Andrew Z. Wang, Alberto A. Gabizon

**Affiliations:** ^1^Shaare Zedek Medical Center, Jerusalem, Israel; ^2^Hadassah-Hebrew University Medical Center, Jerusalem, Israel; ^3^Assuta Medical Center, Tel Aviv, Israel; ^4^Lipomedix Pharmaceuticals Ltd., Jerusalem, Israel; ^5^Department of Radiation Oncology, University of North Carolina-School of Medicine at Chapel Hill, Chapel Hill, NC, United States; ^6^Department of Oncology, Hebrew University-School of Medicine, Jerusalem, Israel

**Keywords:** colorectal cancer, oligometastases, liposomes, mitomycin-C, prodrug, radiotherapy, radiosensitizer

## Abstract

Hypo-fractionated radiotherapy and stereotactic body radiotherapy are viable options for treatment of oligometastases. A prodrug of mitomycin-C is under clinical testing as a pegylated liposomal formulation (Promitil) with an improved safety profile over mitomycin-C. Promitil was offered to two patients with oligometastases from colorectal cancer as radiosensitizer. Each derived durable clinical benefit from Promitil administered immediately prior to and following irradiation. Transient toxicity to normal tissues of moderate to severe degree was observed. Promitil appears to have potential clinical value in this setting.

**HIGHLIGHTS**

- Delivery of radio-sensitizing drugs with pegylated (long-circulating) liposomes is a pharmacologically rational approach which remains largely clinically untested.
- A mitomycin-c prodrug delivered by pegylated liposomes (Promitil) is activated by thiol groups, which are produced in excess by radiation-damaged cells, thus potentiating the radio-sensitizing effect of Promitil.
- Two durable clinical responses in patient with colorectal oligometastases to Promitil and radiotherapy suggest that this approach may be of value in cancer chemo-radiotherapy.

## Introduction

Colorectal cancer (CRC) is the third most commonly diagnosed cancer in the USA (1). During recent decades, advances in surgical technique, diagnostics, and new oncologic drugs have improved outcomes in metastatic disease. Radiation therapy may be used in metastatic colon cancer for palliation at the site of primary tumor or for metastatic lesions. Radiotherapy is effective for palliation in pelvic recurrence of rectal cancer (2, 3). Although hypofractionated treatments may correlate with a higher risk of toxicity, careful selection of palliative patients minimizes those risks (4).

Adding chemotherapy to radiation can increase the anti-tumor effect of radiotherapy. Mitomycin-C (MMC) is a particularly attractive candidate for radiosensitization since it may target the hypoxic population of tumor cells which are considered to be relatively resistant to radiation when compared to well oxygenated cells (5). Promitil is a pegylated liposomal formulation of a lipidated prodrug form of mitomycin C (abbreviated as MLP), developed by Gabizon et al. (6). Promitil reduces MMC toxicity (7), and retains activity against multidrug resistant tumors (8, 9). Liposomes, as other nanoparticles and macromolecules, preferentially accumulate in tumors as a result of the enhanced permeability and retention effect (10). In a recent study (11), we have shown that radiation enhances MMC release from Promitil *in vitro*. Released MMC will sensitize further tumor cells to radiation damage. This background information on Promitil led us to hypothesize that Promitil may be an attractive therapeutic option in palliative therapy of patients with oligometastases treated with radiotherapy.

## Methods

Between 2015 and 2017, Promitil was given immediately prior and following RT to five patients with advanced cancer under individually-named patient compassionate approvals of the Israel Ministry of Health. We focus here on two of these patients^1^, who suffered from advanced CRC with oligometastatic disease confined to active disease in retroperitoneal and pelvic lymph nodes. Both patients gave written informed consent to have their clinical history cases published. All Promitil treatments were given at Shaare Zedek Medical Center (SZMC) at dose levels between 1.0 and 2.0 mg/kg body weight. Promitil was infused as previously described (7), while radiotherapy was delivered at Hadassah Medical Center using Truebeam STX linear accelerator with daily on-board cone beam CT scan image guidance.

## Results

**Figure 1:**
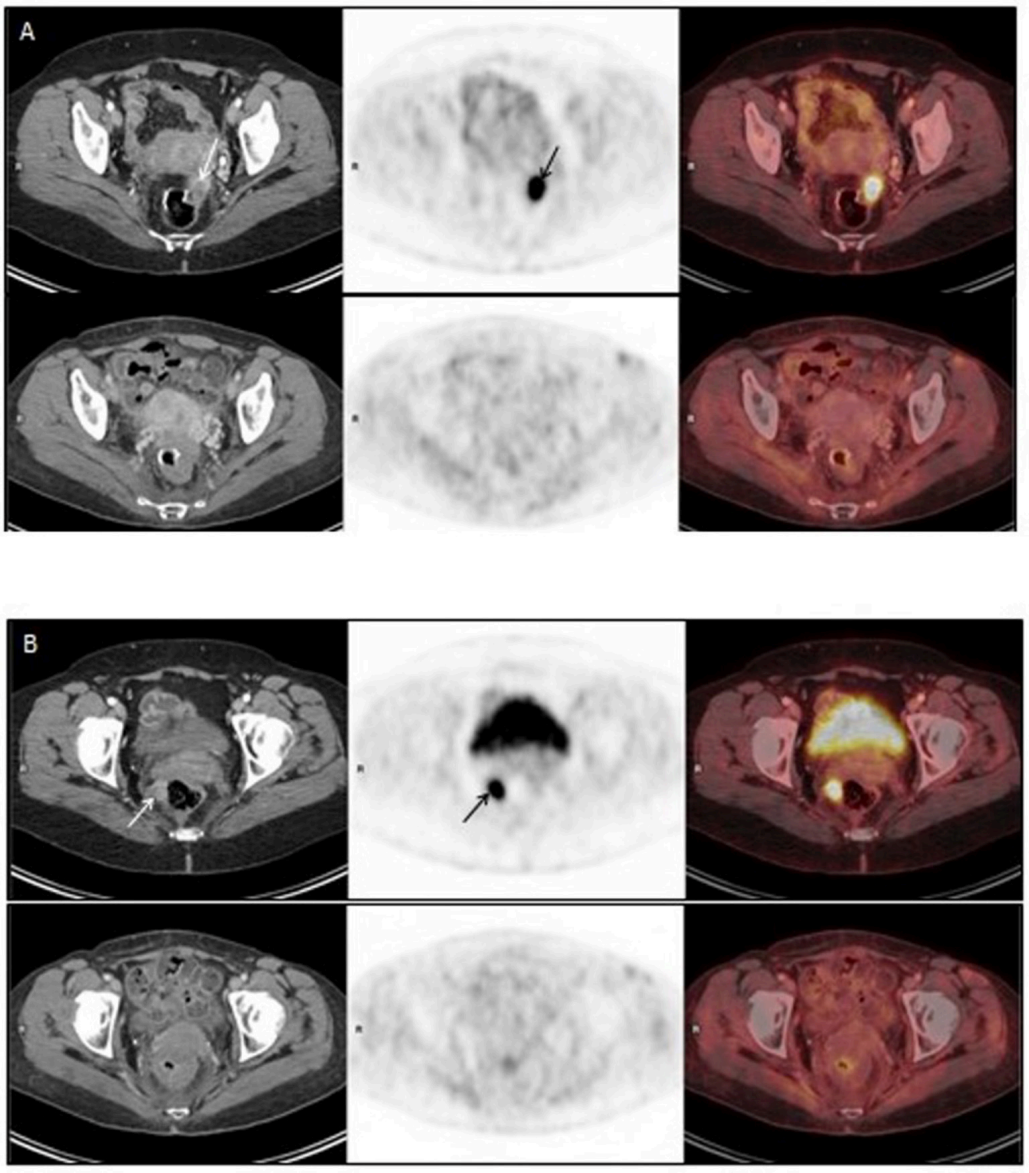
**(A,B)** represent PET-CT images of two different pelvic metastases in the radiation field. Upper panel of **(A,B)**: PET/CT on 30Sep2015, before Promitil with radiotherapy. Lower panel of **(A,B)**: PET/CT on 28Dec2016, 15 months after Promitil with radiotherapy. Axial CT (left panel), FDG PET (middle panel) and fused FDG PET/CT (right panel) images of two metastatic lesions. The intense pathological uptake in a left nodule anteriorly to the rectal anastomosis (**A**, arrows) and in a right para-rectal nodule (**B**, arrows) has completely resolved with therapy on both PET and CT, along a prolonged follow-up (**A**,**B**, lower panel). Note post radiation rectal wall thickening on post-therapy CT images.

**Figure 2:**
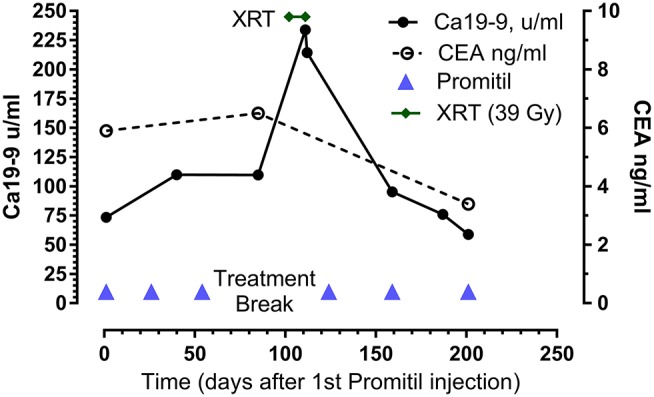
Tumor marker response to Promitil with radiotherapy. Same patient as in Figure 1. Note the sustained decrease of CEA and Ca19-9 levels after chemo-radiotherapy.

The first case is a 58-year old woman, diagnosed in June 2011 with stage 4 rectal adenocarcinoma, RAS-mutant type. On initial diagnosis, she had a rectal tumor and a single liver metastasis which were surgically resected by low anterior resection and partial hepatectomy, followed by a 4-month course of the FOLFOX-bevacizumab regime (9 cycles). In 2013 she developed a single metastasis in the lung, which was treated by SBRT (50 Gy in 5 fractions of 10 Gy), and shortly thereafter retroperitoneal lymphadenopathy. She was treated with standard fractionated radiotherapy to a small retroperitoneal field (42 Gy in 21 fractions of 2 Gy) and chemotherapy was resumed. Between June 2013 and June 2015, she was treated on and off with the FOLFIRI-bevacizumab regime. After further disease progression of lymphadenopathy in the pelvis (in distal location to previous radiation field), suspected recurrence in the liver, and a rise of tumor markers, she was enrolled into a phase 1 clinical trial with a combination of Promitil and capecitabine. After 3 cycles, there was no response by CT scan. Because of increasing pelvic pain, we offered the patient to continue with Promitil, off study, and give palliative radiotherapy for the pelvic recurrence (39 Gy in 13 fractions of 3 Gy delivered in October 2015). She went on to receive 4 more cycles of Promitil together with bevacizumab. The treatment resulted in significant pain relief, complete regression of affected nodes by FDG PET-CT (Figure 1) and a significant and durable drop of tumor marker levels (Figure 2). However, 3–4 months later, the patient developed painful hemorrhagic proctitis requiring weekly blood transfusions. There was a slow and gradual symptomatic improvement with less bleeding, yet, the patient chose to undergo a palliative abdomino-perineal resection and colostomy in April 2017. There was no evidence of residual tumor in the pelvis intra-operatively, nor in the surgical pathological specimen. FDG PET/CT in December 2016 showed a single focus of active disease in the liver. In September 2017, she had IMRT to the liver metastasis (33 Gy in 11 fractions of 3 Gy), but no further treatment with Promitil. Following her last course of Promitil in March 2016, she was without chemotherapy for nearly 2 years. Recently, she resumed chemotherapy due to systemic disease progression in another medical center. She is now surviving 34 months since her first exposure to Promitil and RT.

**Figure 3:**
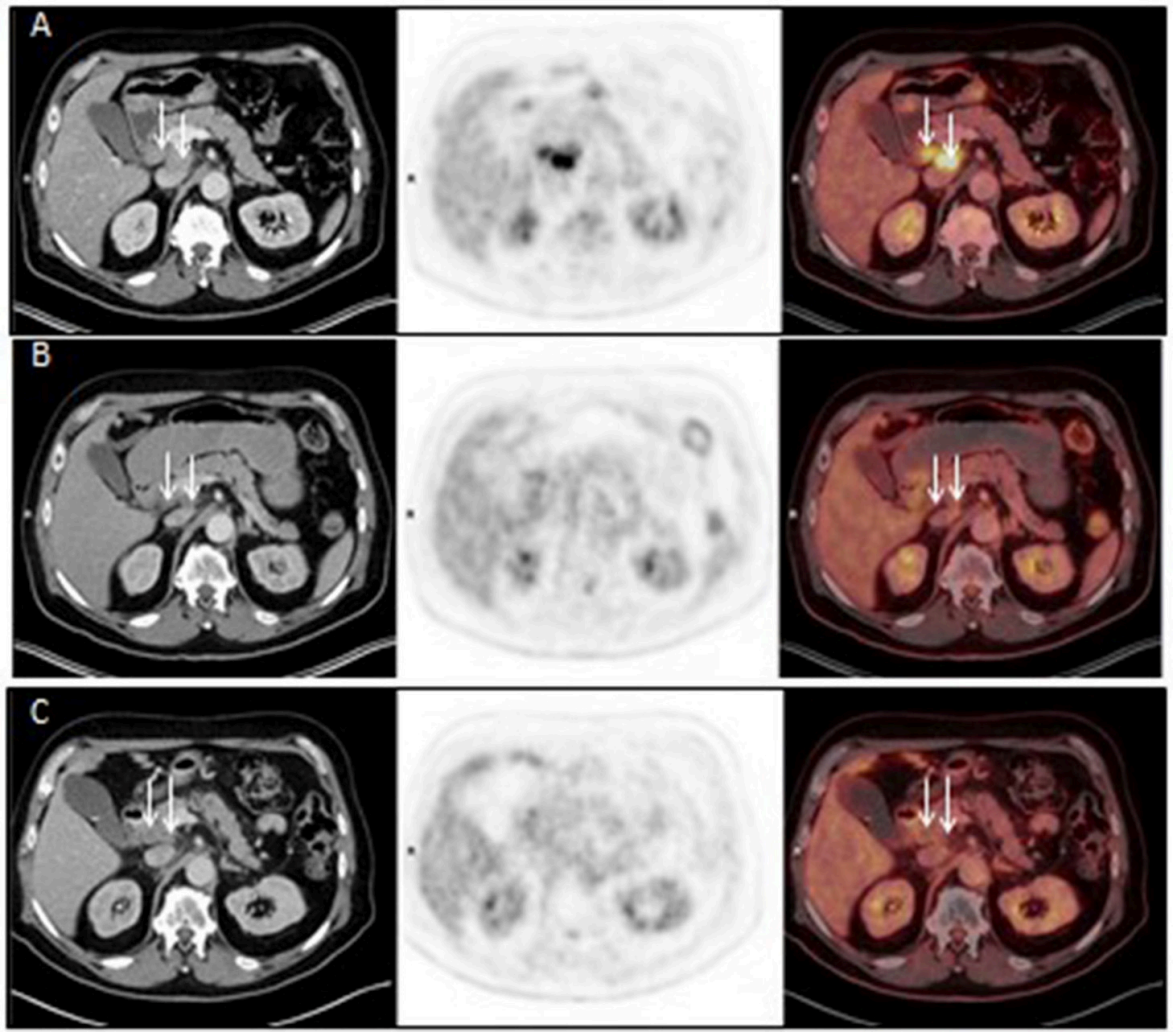
Axial CT (left panel), FDG PET (middle panel) and fused FDG PET/CT (right panel) images before Promitil with radiotherapy (**A**, 07Apr2016), 3 months after Promitil with radiotherapy (**B**, 04July2016), and on a recent re-evaluation 22 months later (**C**, 19Feb2018). Initial pre-therapy intense pathological uptake in two adjacent porto-caval lymph nodes (arrows) resolved after therapy, with significant interval reduction in lymph nodes dimensions.

The second patient is a 67-year old male, diagnosed in February 2013 with T3N1 colon cancer, RAS wild-type, who underwent Lt. hemicolectomy (March 2013), and had adjuvant capecitabine and oxaliplatin. In March 2014, he recurred in a solitary mesenteric node which was surgically removed followed by a second round of adjuvant treatment with bevacizumab and capecitabine. In February 2015, he developed retroperitoneal lymph node metastases, and received single agent cetuximab until January-2016, when disease progression was noted in a group of porto-caval lymph nodes near the hepatic hilum. Cetuximab was discontinued and a combination of irinotecan-bevacizumab was given. However, there was no tumor response, and he was referred for standard fractionated radiotherapy on May 2016 (30 Gy in 10 fractions of 3 Gy) concomitantly with compassionate use of Promitil (5 cycles), which was given along with bevacizumab. He responded with a metabolic CR of the porto-caval hilar lymphadenopathy by FDG PET/CT (Figure 3). In November 2016, following upper abdominal pain, endoscopy revealed a radiation-induced ulcer in the duodenum, which healed slowly but completely with medical treatment (proton pump inhibitors). In September 2017, another group of retroperitoneal lymph nodes in the left para-aortic chain grew. The patient was referred for chemoradiotherapy with Promitil and received protracted retroperitoneal lymph nodes radiotherapy (44 Gy in 22 fractions of 2 Gy to paraaortic nodes and additional 10 Gy in 5 fractions of 2 Gy as boost to the involved nodes) with 2 more cycles of Promitil. For the last 6 months, he remains asymptomatic with ECOG performance status 0 and has been without any active treatment. His last FDG PET/CT shows persistent metabolic response in porto-caval nodes, size reduction of some of the irradiated lymph nodes, and overall mixed response of this retroperitoneal disease. His disease remains confined to abdominal lymph nodes with no visceral spread, and he is now surviving 27 months after first exposure to Promitil-RT.

## Discussion

While early stages of CRC have a relatively good prognosis, and many patients can be cured by surgery alone, the 5-year survival rate declines to 14% for patients diagnosed with metastatic disease. Hellman and Weichselbaum (12, 13) suggested that, in various cancer types, there is an initial oligometastatic phase characterized by the presence of isolated metastases, followed by a second metastatic phase typified by widespread dissemination. The term oligometastases indicates an intermediate state of few metastatic sites and low disease burden in the transition between loco-regional disease and widespread metastases.

Although systemic therapy represents the backbone of metastatic colorectal cancer management, surgical resection in selected patients with oligometastases has been shown to prolong survival, as observed for hepatic (14) and pulmonary (15) metastases. In a population-based study with 13,599 patients from SEER^2^ data, the 5-year overall survival (OS) was 32.8% and 10.5% among patients who did or did not undergo resection of hepatic metastases, respectively (*p* < 0.0001) (16). Far less data are available regarding surgery for less frequent sites of metastases, for example adrenal, ovarian, and retroperitoneal sites. There are no randomized data to strongly support surgical or locally ablative approaches in these scenarios.

Hypofractionated RT is an adequate option for palliative treatment of metastases and may also be effective in control of oligometastatic disease (17). Furthermore, stereotactic body radiotherapy (SBRT)^3^ provides high rates of local control with minimal morbidity for oligometastatic disease and delivers a significantly higher biologically equivalent dose compared to conventional regimens. Two-year local control rates following SBRT for hepatic and pulmonary oligometastases of CRC are ~80% for patients treated with high-dose regimens (18). Retrospective studies have indicated that SBRT for various metastatic lesions results in good outcomes with low morbidity, both in the curative and palliative setting (19–21). Yet, most strategies utilizing radiation with concurrent chemotherapy are still conducted in the setting of conventionally-fractionated radiation therapy.

Adding chemotherapy to radiation can increase the anti-tumor effect of radiotherapy. This is standard therapy in the neoadjuvant setting for rectal adenocarcinoma and for the definitive therapy of tumors of the esophagus, head and neck, anus, as well as uterine cervix and specific stages of gastric and non-small cell lung cancer. Mitomycin C (MMC) is a well-known radiosensitizer. As a DNA crosslinking agent, MMC forms DNA adducts that hinder the ability of cells to repair radiation induced DNA breaks (22), thus increasing the anti-tumor effect. A landmark phase-III trial showed that adding MMC to radiation in the treatment of anal cancer led to better colostomy-free survival and disease-free survival and was also associated with improved 5-year overall survival (78.3 vs. 70.7%, *p* = 0.026) over neoadjuvant cisplatin and 5-FU followed by chemoradiation with cisplatin (23). MMC is a particularly attractive candidate for radiosensitization since it may target the hypoxic population of tumor cells which are considered to be relatively resistant to radiation when compared to well oxygenated cells (5).

Promitil reduces MMC toxicity as shown in humans in a phase 1 study (7) and retains activity against multidrug resistant (MDR-1 type) tumors (8, 9). Its pharmaceutical ingredient is MLP, a prodrug of MMC, which consists of a conjugate of MMC linked to glycerol lipids through a cleavable dithiobenzyl bridge and requires cleavage of the disulfide bond by reducing agents for conversion of the inactive MLP prodrug to active MMC. The MLP prodrug is entrapped in the lipid bilayer of long-circulating pegylated liposomes of similar composition to that of the well-known Doxil/Caelyx formulation (24, 25). Liposomes, as other nanoparticles, preferentially accumulate in tumors as a result of the enhanced permeability and retention (EPR) effect (10). Reducing agents are found in high concentrations within tumors (26).

**Figure 4:**
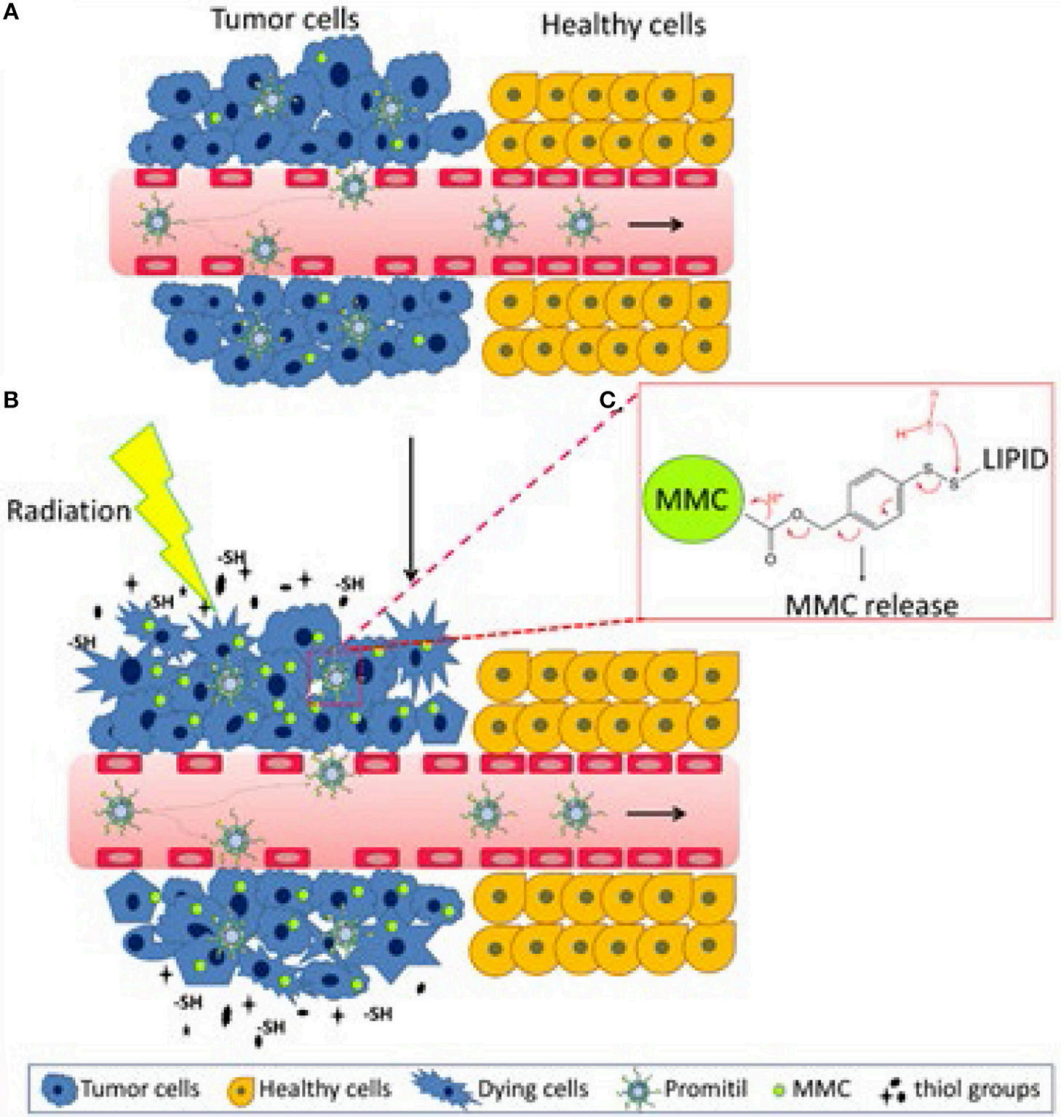
Graphical representation of the proposed mechanism of radio-sensitization by Promitil. The process includes the following steps in sequence. **(A)** Promitil reaches tumor site via EPR effect, limited prodrug activation and MMC release takes place in tumor initiating the radio-sensitizing effect. **(B)** Radiation-damaged cells secrete SH compounds that activate the prodrug MLP amplifying the generation of MMC and subsequent radiosensitizing effect by alkylation and formation of DNA adducts. **(C)** Schematic drawing of the prodrug MLP with the disulfide bridge sensitive to free thiols and leading to the release of MMC. Adapted with permission from Tian et al. (11).

In a recent study (11), we demonstrated that radiation enhances MMC release from Promitil *in vitro* by increasing the levels of thiol-reducing agents secreted from radiation-damaged tumor cells to the surrounding medium. Released MMC, in turn, will sensitize further tumor cells to radiation lethal damage. This bidirectional interaction of radiation and Promitil will conceivably enhance the synergy and final therapeutic efficacy of this combination, particularly if we consider the EPR effect which will contribute to high accumulation of Promitil liposomes in the tumor bed. A graphical depiction of this mechanism of action is presented in Figure 4.

Indeed, *in vivo* studies in human tumor models of colon cancer indicate a superior anti-tumor effect of Promitil and radiotherapy (RT) over MMC or 5FU and RT (11). In this study, a single injection of Promitil potently sensitized colorectal tumor xenografts to fractionated RT; however, a single injection of equitoxic free MMC with or without 5-FU did not. In addition, animals treated with Promitil could receive more than twice the equivalent dose of MMC than animals in the free MMC group because of the reduced toxicity of Promitil, thus conferring an additional pharmacological advantage to the combination of Promitil and RT.

This background information on Promitil led us to hypothesize that Promitil may be an attractive therapeutic option in palliative therapy of patients with oligometastases. Herein, we report two patients with heavily pretreated metastatic colorectal cancer and a clinical course characterized by a persistent oligometastatic phase and a major and lasting clinical benefit after treatment with Promitil and RT.

While the presence of any type of metastases from most solid tumors are generally regarded as being representative of disseminated cancer and are not considered to be curable, evidence has emerged that the subgroup of patients with oligometastases can be cured or at least palliated for long periods of time by resection or ablation of these lesions. This theory provides a rationale for pursuing aggressive local management of oligometastases in well selected CRC patients.

Pharmacokinetic advantages of Promitil include increased circulation time of the prodrug vs. free MMC (t$\frac{1}{2}$ in humans ~24 h for prodrug and <0.5 h for MMC), enhanced accumulation within tumors by the EPR effect, and controlled sustained release depending on the rate of prodrug activation and/or liposome breakdown. Extended intra-tumoral release of activated prodrug from nanoparticles may be particularly important in fractionated radiation schedules to enable synergistic effects.

Promitil has reduced systemic toxicity when the dose of its active ingredient, MLP, is compared to molar-equivalent doses of free MMC in animals and in humans (7, 27). This feature is probably related to various factors. First, pegylated liposomes are very stable and leakage in plasma of MLP prodrug or cleavage to MMC is negligible (7, 27). Second, tissue distribution of the liposomal prodrug may relatively spare some tissues (e.g., kidney, lung, bone marrow) that are sensitive to MMC damage. Third, prodrug cleavage and release of MMC occur gradually, thereby reducing the damage of acute exposure.

It should be noted that the two patients described here developed radiation-induced hemorrhagic proctitis and duodenal ulcer, respectively. This suggests a radiation-enhancing effect of Promitil, but it is also a warning of potential toxicities on normal tissues of this potent combination. These are worrisome complications for the safety profile of this combination although, in the proctitis case, bevacizumab treatment may have contributed to toxicity by inhibiting tissue repair. As mentioned in Methods, we treated another 3, non-CRC, patients with Promitil and RT. In none of these patients we observed toxicity to normal tissues. Two of these patients with widespread metastases of urinary tract cancer died within 6 months after RT. The 3rd patient with locally advanced pancreatic cancer responded extremely well and is alive 16 months after RT with local control in the irradiated site but tumor outgrowth outside the field margins. At any rate, given some concern for increased normal tissue toxicity, close attention to the technique, dose, and fractionation of radiotherapy should be paid in future trials of RT with Promitil. In summary, the triggered release of Promitil by radiation combines the pharmacologic benefits of rapid drug onset and prolonged drug release, making it a potent radiosensitizer.

These are the first reported cases of RT given with Promitil treatment. Based on these encouraging clinical cases, and on the strong preclinical rationale, Promitil is an attractive tool for chemoradiotherapy of patients with CRC oligometastases. To confirm these observations, a phase 1B clinical study to explore further the combined activity of Promitil and RT in palliative treatment of patients with advanced and/or metastatic disease has been recently launched.

## Ethics statement

The cases presented in this study were treated under the compassionate named patient approval procedure of the Israel Ministry of Health. All subjects gave written informed consent for treatment in accordance with the Declaration of Helsinki and our hospital ethical regulations. We confirm that any aspect of the work covered in this manuscript that has involved human patients has been conducted with the ethical approval of all relevant bodies.

## Author contributions

ET drafting the work and revising it for important intellectual content, acquisition, analysis, and interpretation of data. RB-S acquisition, analysis and interpretation of data. ES acquisition, analysis, and interpretation of data. RP and BWC revising the work for important intellectual content. IN, YT, and MG acquisition of data. PO revising the work for important intellectual content. AZW conception of the work, revising the work for important intellectual content. AAG conception and design of the work, acquisition, analysis, and interpretation of data, revising the work for important intellectual content, provide approval for publication of the content, agree to be accountable for all aspects of the work in ensuring that questions related to the accuracy or integrity of any part of the work are appropriately investigated and resolved.

### Conflict of interest statement

AG is Director of Lipomedix Pharm. Ltd., the company providing the experimental drug product Promitil described in this article. PO is VP of Medical Affairs at Lipomedix Pharm. Ltd. The remaining authors declare that the research was conducted in the absence of any commercial or financial relationships that could be construed as a potential conflict of interest.

## Footnotes

^1^The other 3 patients are: 2 patients with urinary tract cancer and widespread metastases who received Promitil and RT for palliation of painful metastases, and 1 patient with locally advanced pancreatic cancer after failure to Folfirinox chemotherapy who received Promitil and SBRT.

^2^Surveillance, Epidemiology, and End Results Program (SEER) Program of the National cancer institute, National Institute of Health, US.

^3^SBRT is also known as Stereotactic Ablative Radio-Therapy.
